# Hematological approaches to multiple myeloma: trends from a Brazilian subset of hematologists. A cross-sectional study: Condutas hematológicas perante o mieloma múltiplo: tendências de um subgrupo de hematologistas brasileiros. Um estudo transversal

**DOI:** 10.1590/1516-3180.2015.0223030416

**Published:** 2015-11-13

**Authors:** Lucila Nassif Kerbauy, Simrit Parmar, José Mauro Kutner, Breno Moreno de Gusmão, Nelson Hamerschlak

**Affiliations:** 1 MD. Attending Physician at the Oncology and Hematology Center Família Dayan-Daycoval, Hospital Israelita Albert Einstein, São Paulo, SP, Brazil.; 2 MD, MSCI. Associate Professor of Medicine, Department of Stem Cell Transplant and Cellular Therapy, University of Texas at MD Anderson Cancer Center, Houston, Texas. United States.; 3 MD, PhD. Attending physician at the Oncology and Hematology Center Família Dayan-Daycoval, Hospital Israelita Albert Einstein, São Paulo, SP, Brazil.

**Keywords:** Multiple myeloma, Hematology, Physician's practice patterns, Evidence-based medicine, Evidence-based practice, Mieloma múltiplo, Hematologia, Condutas na prática dos médicos, Medicina baseada em evidências, Prática clínica baseada em evidências

## Abstract

**CONTEXT AND OBJECTIVE:**

For the last nine years, hematologists and oncologists have gathered annually at an educational symposium organized by a Brazilian and an American hospital. During the 2015 Board Review, a survey among the attendees evaluated the differences in management and treatment methods for multiple myeloma (MM).

**DESIGN AND SETTING:**

Cross-sectional study during an educational hematology symposium in São Paulo, Brazil.

**METHODS:**

Hematologists present at the symposium gave responses to an electronic survey by means of mobile phone.

**RESULTS:**

Among the 350 attendees, 217 answered the questionnaire. Most of the participants believed that immunotargeting agents (iTA) might be effective for slowing MM progression in heavily pretreated patients (67%) and that continued exposure to therapy might lead to emergence of resistant clones in patients with MM (76%). Most of the physicians use maintenance therapy after hematopoietic stem cell transplantation (95%) and 45% of them would further restrict it to post-transplantation patients with underlying high-risk disease. The first-line drugs used for transplantation-ineligible patients (TI-MM) were bortezomib-thalidomide-dexamethasone (31%), bortezomib-dexamethasone (28%), lenalidomide-dexamethasone (Rd; 17%) and melphalan-based therapy (10%). Lenalidomide was the drug of choice for post-transplantation maintenance for half of the participants. No significant differences were observed regarding age or length of experience.

**CONCLUSION:**

The treatment choices for TI-MM patients were highly heterogenous and the melphalan-based regimen represented only 10% of the first-line options. Use of maintenance therapy after transplantation was a common choice. Some results from the survey were divergent from the evidence in the literature.

## RESUMO

**CONTEXTO E OBJETIVOS:**

Há nove anos, hematologistas e oncologistas se reúnem anualmente em um simpósio educacional organizado por um hospital brasileiro e outro norte-americano. Durante o Board Review 2015, uma pesquisa foi conduzida entre os participantes e avaliou as diferenças na conduta e opções de tratamento para o mieloma múltiplo (MM).

**DESENHO E LOCAL:**

Estudo transversal no simpósio educacional de hematologia em São Paulo.

**MÉTODOS:**

Hematologistas presentes no simpósio responderam a uma pesquisa por celular.

**RESULTADOS:**

Dos 350 inscritos, 217 responderam o questionário. A maioria dos participantes acredita que a terapia-alvo imune (iTA) pode ser efetiva para desacelerar a progressão do MM em pacientes que já foram muito tratados previamente, e que a exposição contínua à terapia pode gerar clones resistentes em pacientes com MM (76%). A maioria usa terapia de manutenção após transplante de células-tronco hematopoiéticas (95%) e 45% dos médicos a restringiriam a pacientes pós-transplante com doença de base de alto risco. As drogas de primeira linha adotada para os pacientes inelegíveis para transplante (PIT) foram bortezomibe-talidomida-dexametasona (31%), bortezomibe-dexametasona (28%), lenalidomina-dexametasona (Rd; 17%) e terapia baseada em melfalan (10%). A lenalidomida foi a droga de escolha para a manutenção pós-transplante para metade dos participantes. Nenhuma diferença significativa foi encontrada para idade ou tempo de experiência.

**CONCLUSÃO:**

As escolhas de tratamento para PIT foram altamente heterogêneas e o regime baseado em melfalan representou somente 10% das opções de primeira linha. Terapia de manutenção após transplante é opção comum. Alguns dos resultados do levantamento foram divergentes das evidências na literatura.

## INTRODUCTION

Multiple myeloma (MM) is a plasma cell disease that represents about 10% of hematological malignancies and has an annual incidence of up to 5.6 per 100,000 individuals in the western hemisphere.^1^^,^^2^ On a worldwide scale, approximately 86,000 new cases of MM occur annually.^3^ Regarding Latin American epidemiological datasets, little is known about the incidence and clinical features,^3^^,^^4^ and the exact incidence of MM in Brazil has not yet been determined,^4^^,^^5^ but according to the International Myeloma Foundation, there are around 30,000 Brazilian MM patients currently under treatment.^6^ The management of MM has been revolutionized over the last few years and this has been based on understanding recent advances in MM pathophysiology, discovery of new target pathways and development of novel therapeutic agents.^7^

Over the last nine years, Albert Einstein Hospital (São Paulo, SP, Brazil), in collaboration with MD Anderson Cancer Center (Houston, TX, USA), developed an annual ­state-of-the-art hematological symposium that was attended by over 400 hematologists from Latin America (mostly Brazil). In 2015, the symposium was held on June 23-26 and included a hematological review course, which promoted opportunities for physicians (mostly clinical hematologists and oncologists) to update and share their understanding of diseases and to disseminate practical knowledge (including in relation to therapeutic agents) on different topics within hematology. The educational content included both malignant and benign hematology. The majority of the lectures were in Portuguese, and there were four international speakers. In the light of recent advances and controversies, one of the highly appreciated topics discussed was that of MM.

## OBJECTIVES

With the aim of assessing Latin American common standards of care, and their distinctions, and also the experience and expectations of hematologists concerning new treatments, a survey was developed and administered among the Brazilian symposium attendees. The objective of the survey was to evaluate the controversies and differences in practical management and treatment methods for MM. We hypothesized that better understanding of Latin American hematologists' treatment choices would allow us to identify and improve physician support and patient care.

## METHODS

**Figure 1:**
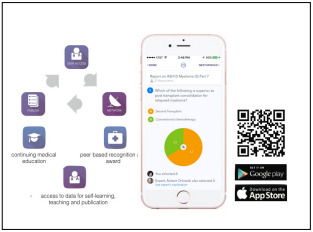
On-screen appearance of MDRing, an application created for surveying the physicians during the symposium.

During the Board Review of the Ninth International Symposium for Updating on Hematological Topics and the Ninth Symposium for Bone Marrow Transplantation (IX Simpósio Internacional de Atualização em Temas de Hematologia/IX Simpósio de Transplante de Medula Óssea), held jointly from June 23 to 26, 2015, 350 participants were invited to answer a survey on MM therapy using a free mobile phone application called MDRing (Figure 1). Questions were synchronized with the lecture topics and the participants were encouraged to answer the questionnaire preferably before each presentation. Each electronic questionnaire had multiple closed options for responses and the numbers of options for each question were variable. In addition, pertinent demographic information was collected, including gender, academic practice, age and number of years of professional experience. The survey was composed of 15 questions involving treatment-related topics, including immunotargeting, transplantation, options for multiple myeloma patients who are ineligible for transplantation and maintenance treatment.

We report here the survey results as percentages of respondents, excluding those who did not provide an answer to a ­particular question. Subgroup analyses were conducted according to gender, age (groups of greater than or equal to 35 years or less than 35 years) and experience (more than 10 years after specialization and less than 10 years). The univariate statistical analysis included the chi-square test. The statistical analyses were performed using the SPSS software (IBM, Chicago, 2013). The significance level was set at a P-value of 0.05.

## RESULTS

**Figure 2:**
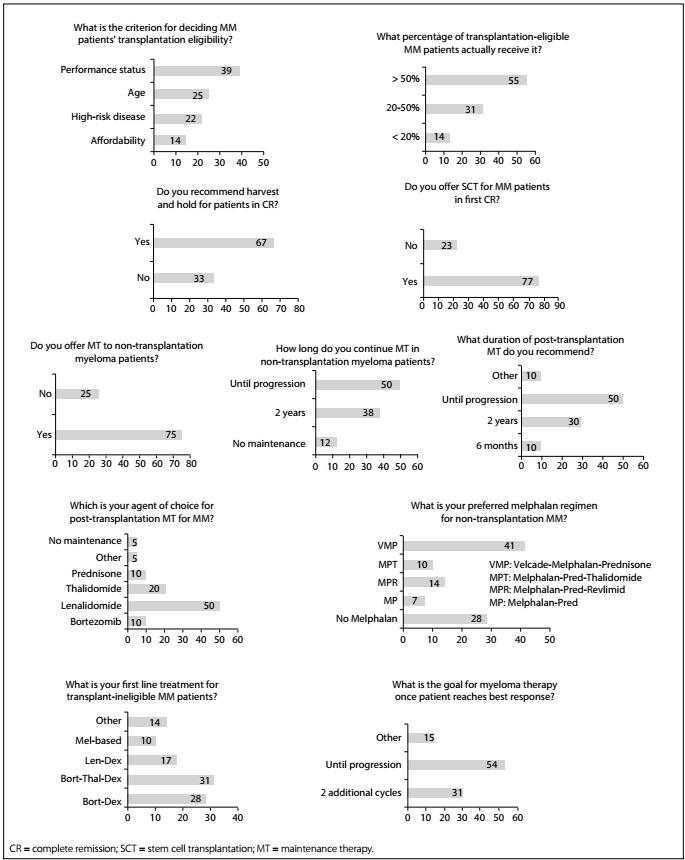
Responses to questions about treatment methods for multiple myeloma (MM) among hematologists attending a symposium in São Paulo, Brazil, in 2015.

During the four-day symposium, a total of 217 participants answered the questionnaire completely. The median age of the population studied was 35 years (range: 25-47 years); 59% were male; the median length of time since graduation was 10 years (range: 3-24 years); and 53% had less than 10 years of experience. The survey participants' characteristics and their responses are shown in Tables 1, ^2^, ^3^ and ^4^ and Figure 2. Although some participants at the symposium were from other Latin American countries, only Brazilian hematologists answered the questionnaire on this occasion.

**Table 1:**
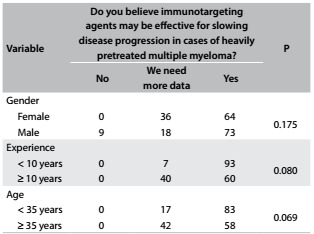
Perceptions about the effectiveness of immunotargeting agents according to age, clinical experience and gender of the respondent (%)

**Table 2:**
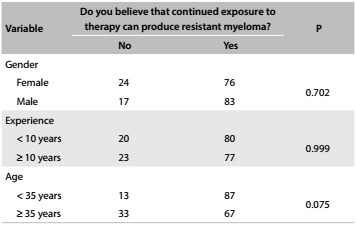
Perceptions about resistance caused by continued exposure to therapy, according to age, clinical experience and gender (%)

**Table 3:**
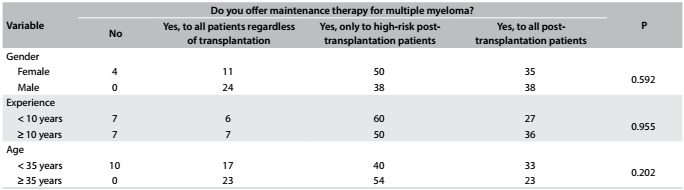
Personal experience with maintenance therapy according to age, experience and gender (%)

**Table 4:**
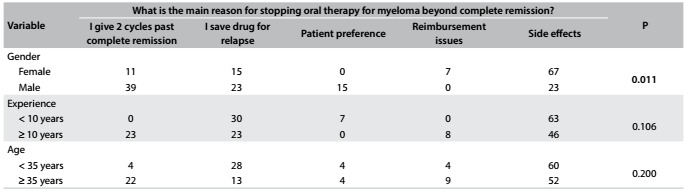
Main reason for stopping oral therapy for multiple myeloma beyond complete remission (%)

In analyzing new drugs, the majority of the participants believed that immunotargeting agents (iTA) might be effective in slowing disease progression in MM patients with multiple lines of prior therapy. Younger physicians (83% versus 58%; P = 0.069) and physicians with less than 10 years of experience (93% versus 60%; P = 0.08) tended to consider that iTA would be effective in this situation, although the difference was not significant. iTA was perceived to be important equally by male and female physicians (73% males versus 64% females; P > 0.175; Table 1).

The majority of the physicians (76%) believed that continued exposure to therapy might lead to emergence of resistant clones in patients with MM. No significant differences were observed based on participant's age, gender or years in practice, for this variable (Table 2).

With regard to maintenance therapy (MT), the majority (95%) of the physicians declared that they would offer it to patients undergoing treatment for MM. However, the majority (46%) would restrict maintenance therapy to post-transplantation cases that were classified as high-risk, while 29% of the physicians would extend MT to all transplantation patients. No significance difference was found in relation to gender, age or years in practice (Table 3).

Side effects were considered to be the main reason (56%) for halting oral therapy for MM beyond complete remission, followed by the practice of saving therapy for future relapses (22%) and the practice of using a fixed drug approach (12%), in which the physician offers two additional cycles beyond complete remission. There was no significant difference in age or years in practice among these respondents (Table 4).

The responses to additional questions on transplantation and maintenance pulse therapy for MM patients are shown in the graphs of Figure 2. Regarding first-line treatment for transplantation-ineligible MM patients, we observed that 31% of the physicians used bortezomib-thalidomide-dexamethasone, 28% bortezomib-dexamethasone and 17% lenalidomide-dexamethasone (Rd), while only 10% of the participants chose melphalan-based therapy. In relation to the melphalan-based regimen of choice for non-transplantation myeloma cases, 41% of the participants chose a regimen with bortezomib, known as bortezomib-melphalan-prednisone (VMP), followed by melphalan-prednisone-lenalidomide (MPR) (14%). Melphalan-prednisone-thalidomide (MPT) was the option for only 10% of the physicians and melphalan-prednisone (MP) for 7%. About half of all the survey respondents answered that they continued to provide maintenance therapy for MM patients who were not eligible for transplantation until progression, whereas 38% chose maintenance therapy for two years and 12% reported that they were not concerned about this treatment.

Regarding the duration of post-transplantation MT, 50% of the physicians said that they would maintain it until disease progression, 30% would use it for two years, 10% would apply it for six months and the other 10% would not agree with the latter options. Lenalidomide was the drug of choice for post-transplantation maintenance for half of the participants, followed by thalidomide (20%) and bortezomib or prednisone (10%).

## DISCUSSION

The data on the responses regarding iTA presented here demonstrated that younger physicians believed more strongly that iTA was the preferred option for decreasing disease progression. Critical new steps in MM management rely on development of second-generation novel agents and the advent of monoclonal antibodies. Various antigens have been implicated as potential therapeutic targets in MM. CD38 is an important immunotherapy target because of its high level of expression in malignant plasma cells and low expression in other cells, as well as being an important modulator of intracellular signaling.^8^ Preliminary results suggest that the use of CD38-targeting antibodies in case of relapsed or refractory MM presents a safe profile and at least a minimal response rate.^9^ Furthermore, an ability to overdrive genetic mutations, with prolonging of the durable response, has been reported.^10^

As described above, most respondents believed that continued exposure to therapy might lead to emergence of resistant clones. The literature shows that chemoresistance patterns can indeed be acquired. One study reported that the mechanism consisted of a situation of coexistence of several clones, in which treatment was able to eradicate the major chemosensitive clone, but not the minor chemoresistant clone, which eventually became the dominant clone with continued treatment and subsequently drove the proliferation.^11^ Continuous exposure to therapy could contribute to this process and, in cases of adverse cytogenetic abnormalities, maintenance therapy has demonstrated lack of efficacy primarily due to the emergence of tumor-resistant clones in patients with prolonged exposure to thalidomide.^12^^,^^13^

We observed that the great majority of respondents would offer MT to their MM patients, and their first-choice drug was lenalidomide. Although a cure for MM is still not possible in many patients, long-term MT can have a positive impact on response duration, progression-free survival and overall survival, assuming controlled minimal toxicity rates, as shown in several studies.^10^ There is evidence supporting lenalidomide as the best candidate for use as MT. Two randomized trials evaluating maintenance therapy using lenalidomide versus placebo following autologous stem cell transplantation (ASCT) have been published, and have demonstrated that use of lenalidomide provides significantly prolonged progression-free survival of two to four years.^14^^,^^15^ The Cancer and Leukemia Group B (CALGB) trial also demonstrated that the group who received induction therapy with lenalidomide obtained an overall survival benefit.^15^

Most participants in the present study reported that high-dose therapy with ASCT was still the standard of care, corresponding to the preferred therapy for patients at their first complete remission, even in the era of novel therapies. The emergence of deep complete remission with novel drugs has led some groups to test new upfront treatments without immediate transplantation.^10^ The criteria for defining eligibility for transplantation were heterogeneous in the present study, as shown in Figure 2. Regarding the percentage of transplantation-eligible MM patients who actually receive this therapy, 45% of the respondents declared that less than 50% of the patients really underwent transplantation. However, the reason for this was not investigated. We believe that this is an issue worth exploring in future investigations. Lack of availability of public healthcare services for transplantation may have been the reason for this.

The first-line treatment for transplantation-ineligible patients was found to be heterogeneous in this study. Several randomized trials have shown that the MPT regimen can delay disease progression and improve overall survival, in comparison with MP.^16^^,^^17^^,^^18^ A meta-analysis on six randomized trials comparing MPT with MP showed that there was an improvement in progression-free survival and overall survival with MPT, but also an increased rate of toxicity.^19^ Based on these studies, MPT has been approved as the standard of care. The phase III VISTA trial demonstrated better overall survival with VMP, in comparison with MP, after five years of follow-up, among patients ineligible for transplantation.^20^ The European Myeloma Network recommendations indicated that MPT and VMP are the preferred regimen for transplantation-ineligible patients.^21^ Indeed, according to a recent review of clinical trials undertaken globally, MPT and VMP are the first-choice regimens for transplantation-ineligible patients.^13^ However, the melphalan-based regimen represented only 10% of the options as first-line treatment for transplantation-ineligible multiple myeloma in our study. Use of bortezomib-thalidomide-dexamethasone (VTD), which was the first choice among our participants, and use of a bortezomib-dexamethasone (VD) regimen alone, which was their second choice for patients with transplantation-ineligible multiple myeloma, were evaluated in the UPFRONT trial, published recently in the *Journal of Clinical Oncology* . These options were not inferior to VMP.^22^ With a median follow-up of 42.7 months, the median progression-free survival, median overall survival and overall response rates were similar for these three options, with no significant difference. Nevertheless, VTD, which was the first choice in Brazil in our study, was correlated with greater numbers of common adverse events than VMP and VD, based on the UPFRONT trial. The results from a multicenter open-label phase III trial (FIRST) comparing the efficacy and safety of Rd versus MPT among transplantation-ineligible patients demonstrated that Rd significantly improved the primary endpoint of progression-free survival, compared with MPT.^23^ Based on these findings, continuous Rd, which was the third treatment option among our physicians, could become a new standard of treatment for these patients.

We believe our survey helps to identify how physicians approach patient care and treatment in multiple myeloma cases and the expectations for the future. We are thus opening a worldwide dialogue about opportunities for improving physician support and patient treatment.

## CONCLUSIONS

The physicians surveyed believed that iTA would be an option for decreasing disease progression among MM patients. These Latin American hematologists mostly adopted MT over the long-term, with lenalidomide as the first-choice drug. The criteria for defining eligibility for stem cell transplantation were quite heterogeneous, according to the hematologists' responses, as also were the criteria regarding first-line treatment for patients who were ineligible for transplantation. This last response was divergent from the evidence in the literature. Evidence-based medical education initiatives are therefore necessary.

## References

[1] Smith A, Howell D, Patmore R, Jack A, Roman E (2011). Incidence of haematological malignancy by sub-type: a report from the Haematological Malignancy Research Network. Br J Cancer.

[2] Palumbo A, Anderson K (2011). Multiple myeloma. N Engl J Med.

[3] Becker N (2011). Epidemiology of multiple myeloma. Recent Results Cancer Res.

[4] Hungria VT, Maiolino A, Martinez G (2008). Confirmation of the utility of the International Staging System and identification of a unique pattern of disease in Brazilian patients with multiple myeloma. Haematologica.

[5] Hungria VTM, Maiolino A, Martinez GA (2013). Multiple myeloma profile in Latin America: clinical and epidemiological observational study. Blood.

[6] Minnicelli C, Maciel JF, Hassan R, Lemos TM (2015). Clinical and epidemiological features of multiple myeloma patients from a low socio-economic region of Brazil. Rev Bras Hematol Hemoter.

[7] Ayed AO, Chang LJ, Moreb JS (2015). Immunotherapy for multiple myeloma: Current status and future directions. Crit Rev Oncol Hematol.

[8] Phipps C, Chen Y, Gopalakrishnan S, Tan D (2015). Daratumumab and its potential in the treatment of multiple myeloma: overview of the preclinical and clinical development. Ther Adv Hematol.

[9] Plesner T, Arkenau HT, Lokhorst HM (2014). Safety and efficacy of daratumumab with lenalidomide and dexamethasone in relapsed or relapsed, refractory multiple myeloma. J Clin Oncol.

[10] Richardson PG, Laubach JP, Munshi NC, Anderson KC (2014). Early or delayed transplantation for multiple myeloma in the era of novel therapy: does one size fit all?. Hematology Am Soc Hematol Educ Program.

[11] San Miguel J. (2014). Multiple myeloma: a model for scientific and clinical progress. Hematology Am Soc Hematol Educ Program.

[12] Morgan GJ, Gregory WM, Davies FE (2012). The role of maintenance thalidomide therapy in multiple myeloma: MRC Myeloma IX results and meta-analysis. Blood.

[13] Moreau P, Attal M, Facon T (2015). Frontline therapy of multiple myeloma. Blood.

[14] Attal M, Lauwers-Cances V, Marit G (2012). Lenalidomide maintenance after stem-cell transplantation for multiple myeloma. N Engl J Med.

[15] McCarthy PL, Owzar K, Hofmeister CC (2012). Lenalidomide after stem-cell transplantation for multiple myeloma. N Engl J Med.

[16] Facon T, Mary JY, Hulin C (2007). Melphalan and prednisone plus thalidomide versus melphalan and prednisone alone or reduced-intensity autologous stem cell transplantation in elderly patients with multiple myeloma (IFM 99-06): a randomised trial. Lancet.

[17] Wijermans P, Schaafsma M, Termorshuizen F (2010). Phase III study of the value of thalidomide added to melphalan plus prednisone in elderly patients with newly diagnosed multiple myeloma: the HOVON 49 Study. J Clin Oncol.

[18] Hulin C, Facon T, Rodon P (2009). Efficacy of melphalan and prednisone plus thalidomide in patients older than 75 years with newly diagnosed multiple myeloma: IFM 01/01 trial. J Clin Oncol.

[19] Fayers PM, Palumbo A, Hulin C (2011). Thalidomide for previously untreated elderly patients with multiple myeloma: meta-analysis of 1685 individual patient data from 6 randomized clinical trials. Blood.

[20] San Miguel JF, Schlag R, Khuageva NK (2013). Persistent overall survival benefit and no increased risk of second malignancies with bortezomib-melphalan-prednisone versus melphalan-prednisone in patients with previously untreated multiple myeloma. J Clin Oncol.

[21] Engelhardt M, Terpos E, Kleber M (2014). European Myeloma Network recommendations on the evaluation and treatment of newly diagnosed patients with multiple myeloma. Haematologica.

[22] Niesvizky R, Flinn IW, Rifkin R (2015). Community-Based Phase IIIB Trial of Three UPFRONT Bortezomib-Based Myeloma Regimens. J Clin Oncol.

[23] Facon T, Dimopoulos MA, Dispenzieri A (2013). Initial phase 3 results of the first (frontline investigation of lenalidomide + dexamethasone versus standard thalidomide) Trial (MM-020/IFM 07 01) in newly diagnosed multiple myeloma (ndmm) patients (PTS) ineligible for stem cell transplantation (SCT). Blood.

